# Cathodal Transcranial Direct Current Stimulation on the Right Temporo-Parietal Junction Modulates the Use of Mitigating Circumstances during Moral Judgments

**DOI:** 10.3389/fnhum.2016.00355

**Published:** 2016-07-12

**Authors:** Laëtitia Leloup, Diana Dongo Miletich, Gaëlle Andriet, Yves Vandermeeren, Dana Samson

**Affiliations:** ^1^Psychological Sciences Research Institute, Université catholique de Louvain, Louvain-la-NeuveBelgium; ^2^NeuroModulation Unit, Neurology Department, CHU UCL Namur, Université catholique de Louvain, YvoirBelgium; ^3^Institute of Neuroscience, Université catholique de Louvain, BrusselsBelgium

**Keywords:** tDCS, rTPJ, moral judgment, moral responsibility, mitigating circumstances

## Abstract

Recently, a few transcranial magnetic stimulation or transcranial direct current stimulation (tDCS) studies have shown that the right temporo-parietal junction (rTPJ) plays a causal role in moral reasoning especially in cases of accidental harms or attempted harms. The profile of results across studies is, however, not entirely consistent: sometimes the stimulation affects predominantly attempted harms while sometimes the stimulation affects predominantly accidental harms. We argue that such discrepancy could reflect different functional contributions of the rTPJ in moral judgments and that the chosen design parameters or stimulation method may differentially bring to light one or the other functional role of the rTPJ. In the current study, we found that tDCS specifically affected accidental harms but not attempted harms. Low cathodal stimulation of the rTPJ led to a marginally significant increase in the severity of judgments of accidental harms (Experiment 1) while higher cathodal current density led to a highly significant decrease in the severity of judgments of accidental harms (Experiment 2). Our pattern of results in the context of our experimental design can best be explained by a causal role of the rTPJ in processing the mitigating circumstances which reduce a protagonist’s moral responsibility. We discuss these results in relation to the idea that the rTPJ may play multiple roles in moral cognition and in relation to methodological aspects related to the use of tDCS.

## Introduction

The right temporo-parietal junction (rTPJ) is seen as one of the key regions of what is now commonly coined the “theory of mind network” (Saxe and Kanwisher, 2003; Young et al., 2010b; Krall et al., 2014; Schurz et al., 2014), i.e., the brain network sustaining our ability to explain and predict someone’s behavior on the basis of his or her mental states (Premack and Woodruff, 1978). More recently, the rTPJ has also been associated with moral reasoning, i.e., when participants are asked what someone ought to do (Greene et al., 2001; Koenigs et al., 2007) or when participants are asked to judge whether what someone is doing is permissible, should be blamed or should be punished (Young et al., 2007; Cushman, 2008; Young and Saxe, 2008; Koster-Hale et al., 2013; Yoder and Decety, 2014).

The majority of the studies which have shown the involvement of the rTPJ in moral cognition are imaging studies which only provide correlational evidence. Only a handful of studies have investigated the potential causal role of the rTPJ in moral cognition. The study by Jeurissen et al. (2014) examined moral judgments in the context of moral dilemmas, emphasizing thus the role of emotions in moral judgments while three other studies (Young et al., 2010a; Sellaro et al., 2015; Ye et al., 2015) examined moral judgments in the context of accidental harm or failed attempts to harm (hereafter referred to as attempted harm), examining thus the role of intentional attribution and theory of mind in moral judgments.

In the paradigm used in these three latter studies (Young et al., 2010a; Sellaro et al., 2015; Ye et al., 2015), the intention of the agent (no intention to harm vs. intention to harm) was orthogonally manipulated with the outcome of the action (neutral outcome vs. harmful outcome) to create four types of scenarios: two types of scenarios where the intention and the outcome are compatible (“neutral scenarios” where the protagonist has no intention to harm and no harm occurs as outcome of the action or “intentional harm scenarios” where the intention is to harm and harm occurs as outcome of the action) and two types of scenarios in which the intention and the outcome are in conflict (“accidental harm scenarios” in which the agent has no intention to harm but an harmful outcome occurs and “attempted harm scenarios” in which the agent intends to harm but no harmful outcome occurs).

Interestingly, rTPJ stimulation modulated judgments of certain moral scenarios more specifically and not always the same type of scenarios across studies (see **Table 1**). Understanding the origin of these discrepancies could provide useful insights into the role of the rTPJ in moral judgments. If the role of the rTPJ in moral judgment is related to the attribution of mental states, we should expect that the effects of rTPJ stimulation will directly depend on the mentalizing demands of the moral scenarios. Such demands may vary not only quantitatively (some scenarios requiring more mentalizing than others) but also qualitatively (different scenarios leading to different forms of mentalizing). At least three qualitatively different sources of mentalizing demands can be identified in the moral scenarios described above. One source of mentalizing demands relates to the processing of the agent’s belief about the outcome of his action. It has been extensively documented in the theory of mind literature that the rTPJ is particularly sensitive to situations where an agent holds a false belief (e.g., Saxe and Kanwisher, 2003; Perner et al., 2006), that is, when there is a mismatch between the agent’s perspective (what the agent falsely thinks about the state of the world) and the participant’s perspective (what the participant knows is the true state of the world). According to this interpretation of the role of the rTPJ, one would expect the rTPJ stimulation to only affect moral judgments in the attempted harm and accidental harm scenarios. Indeed, these are the only two conditions in which the agent of the story holds a false belief (the mismatch between the intention and the outcome in these conditions always resulted from the agent holding a false belief about the potential outcome of his action). None of the transcranial magnetic stimulation (TMS) or transcranial direct current stimulation (tDCS) results perfectly fit with this interpretation (**Table 1**) even though this seems to be the main interpretation put forward in these studies. In the case of Young et al. (2010a) and Ye et al. (2015), a significant effect of stimulation was found for attempted harm scenarios but the effect did not reach significance for accidental harm scenarios. Sellaro et al. (2015) found the opposite profile, with an effect for accidental harm scenarios but not for attempted harm scenarios. It has been proposed that this discrepancy simply results from ceiling effects which lead to a lack of sensitivity to inhibitory stimulation when one judges accidental harms and a lack of sensitivity to excitatory stimulation when one judges attempted harms (Sellaro et al., 2015). Indeed, in their article, Sellaro et al. (2015) explained their diverging results from Young et al. (2010a) with the idea that belief information has a different weight in the accidental and attempted harm scenarios. More specifically, the negative belief information leading to the understanding that the agent has a desire to harm someone else is salient in the attempted harm scenarios and so the activation of the rTPJ is claimed to be more pronounced. In the case of the accidental harm scenarios, the neutral belief information is less salient and the activation of the rTPJ is claimed to be less pronounced. Increasing the excitability of the rTPJ would thus be more likely to increase the contribution of belief representations in the case of the accidental harm scenarios (where the rTPJ is less activated because of the neutral intention) than in the case of the attempted harm scenarios (where the rTPJ is already activated because of the harmful intention). The reverse reasoning is applied to the inhibitory stimulation which would be more likely to decrease the contribution of belief representations in the attempted harm scenarios (where the rTPJ is more activated because of the harmful intention) than in the accidental harm scenarios (where the rTPJ is less activated because of the neutral intention).

**Table 1:** Summary of previously reported effects of rTPJ stimulation on moral judgments.

	Type of TPJ stimulation	Question asked	Stimulation parameters	Intentional harm scenarios (intention to harm and harmful outcome)	Attempted harm scenarios (intention to harm but no harmful outcome)	Accidental harm scenarios (no intention to harm but harmful outcome)	Neutral scenarios (no intention to harm and no harmful outcome)
Young et al., 2010a	Inhibited rTPJ (TMS)	“The action was:” on a scale from 1 (Forbidden) to 7 (Permissible).	Experiment 1: oﬄine TMS at 1 Hz for 25’ Experiment 2: online TMS short bursts at 10 Hz for 500 ms	No effect	Judged as more permissible	No effect	No effect
Ye et al., 2015	Decreased excitability of rTPJ (cathodal tDCS) and simultaneously increased excitability of lTPJ (anodal tDCS)	“The action was:” on a scale from 1 (Permissible) to 10 (Forbidden).	**Stimulation electrode** **Size:** 35 cm^2^ **Location:** CP6 **Return electrode** **Size:** 35 cm^2^ **Location:** CP5 **Intensity:** 2 mA **Duration:** 20’ (15’ oﬄine – 5” online) **Current density:** 0.057 mA/cm^2^ **Total charge:** 0.069 C/cm^2^*	No effect	Judged as more permissible	No effect	Judged as more permissible
	Increased excitability of rTPJ (anodal tDCS) and simultaneously decreased excitability of lTPJ (cathodal tDCS)			Judged as less permissible	No effect	No effect	No effect
Sellaro et al., 2015	Decreased excitability of rTPJ (cathodal tDCS) and simultaneously increased excitability of left DLPF cortex (anodal tDCS)	“The action was:” on a scale from 1 (Morally forbidden) to 7 (Morally permissible)	**Stimulation electrode** **Size:** 35 cm^2^ **Location:** CP6 **Return electrode** **Size:** 35 cm^2^ **Location:** left supraorbital area **Intensity:** 1 mA **Duration:** 20’ (oﬄine) **Current density:** 0.029 mA/cm^2^ **Total charge:** 0.034 C/cm^2^*	No effect	No effect	No effect	No effect
	Increased excitability of rTPJ (anodal tDCS) and simultaneously decreased excitability of left DLPF cortex (cathodal tDCS)			No effect	No effect	Judged as more permissible	No effect

*Total charge has been calculated according to the formula used by Nitsche et al. (2003a): [intensity (A)/electrode size (cm^2^)] × total stimulation duration (s).

Beliefs are not the only mental states which mismatch across the participants and the agent presented in the moral scenarios. On the assumption that most people do not want to harm others, there is also a mismatch when the agent has a desire to harm someone. The processing of the agent’s desire may thus be a second source of variability in terms of mentalizing demands across the moral scenarios. This is compatible with evidence for a more general role of the rTPJ in dealing with conflicting mental states (Saxe and Wexler, 2005; Santiesteban et al., 2012). According to this interpretation, we could expect a stronger modulation of moral judgments following rTPJ stimulation when the agent has a desire to harm (i.e., in the case of attempted harm scenarios and intentional harm scenarios) than when the agent has no desire to harm (i.e., in the accidental harm scenarios and in the neutral scenarios). This interpretation of the role of the rTPJ should not be seen as incompatible with a role in belief processing and one could thus expect additive effects of rTPJ stimulation whereby (1) accidental and attempted harm scenarios would be more affected than neutral or intentional harm scenarios because the processing of the agent’s belief and (2) attempted harm scenarios would be even further affected because of the processing of the agent’s harmful desire. Such additive effects are globally compatible with the results of Young et al. (2010a) and those of Ye et al. (2015).

Finally, a third source of variable mentalizing demands in the moral scenarios used so far relates to the processing of mitigating circumstances disculpating the author of the harmful act [for example, when the harm was due to mental illness (Buckholtz et al., 2008, 2015; Koster-Hale et al., 2013)]. According to this account, the rTPJ would play a key role in processing the various pieces of information necessary to judge someone’s moral responsibility (Buckholtz et al., 2015). In the moral scenarios discussed here, only the accidental harm condition involves mitigating circumstances which should affect the agent’s moral responsibility. Indeed, in that condition, while the agent had a causal role in the harmful consequences of his actions, he had no intention to harm. Note that in the case of failed attempts to harm, there is no real mitigating circumstance about the agent’s moral responsibility *per se* (the harmful intention is clear and there are no mitigating circumstances provided in the scenarios to justify the agent’s harmful intention) but there are, however, mitigating circumstances related to the amount of deserved punishment, since no actual harm occurred. The integration of information about the degree of harm caused in order to assign punishment has been hypothesized to be sustained by other brain areas than the rTPJ such as the ventromedial prefrontal cortex (vmPFC), the posterior cingulate cortex (PCC), and the dorsolateral prefrontal cortex (DLPFC; Buckholtz et al., 2008, 2015; Buckholtz and Marois, 2012). The results of Sellaro et al. (2015) which showed that increasing the excitability of the rTPJ with anodal tDCS caused participants to judge accidental harms as more morally permissible with no effect on attempted harms, are in line with this interpretation of the role of the rTPJ in processing mitigating circumstances (even though this is not the authors’ interpretation of their results).

Given that there is independent evidence to support all three roles of the rTPJ in moral reasoning, it is plausible that the specifics of the moral scenarios and moral questions used can accentuate one or the other contributing role of the rTPJ and hence influence the specific pattern of results across the different types of moral scenarios. To our knowledge, the hypothesis that the rTPJ plays a “causal” role in processing the mitigating circumstances which reduce an agent’s moral responsibility has not been directly tested yet. In the current tDCS study, we used a design that should bring out such contributing role of the rTPJ. We used a blame (Experiment 1) or punishment (Experiment 2) question instead of the moral permissibility question used in the previous TMS and tDCS studies. Indeed, judging how much blame or punishment an agent deserves is thought to more directly assess moral responsibility reasoning (e.g., Buckholtz et al., 2008, 2015; Buckholtz and Marois, 2012).

In addition to the change of moral question, we also used a different electrode montage compared to the two previous tDCS studies (Sellaro et al., 2015; Ye et al., 2015) discussed before so that we could more selectively target the rTPJ. In the two previous tDCS studies, a return rather than a reference electrode was used. A return electrode is smaller than a reference electrode and is still considered as an “active” electrode which influences the excitability of the brain region stimulated. In the case of the study by Sellaro et al. (2015), this means that both the rTPJ (where the stimulation electrode was placed) and the left supraorbital area (where the return electrode was placed) were stimulated simultaneously (note, however, that the authors showed that stimulating the left supraorbital area in a montage using a larger reference electrode on the rTPJ was not sufficient to produce the effect on accidental harm, providing thus indirect evidence that it was the stimulation of the rTPJ which was at the origin of the effect). In the case of Ye et al. (2015), it was the left TPJ that was stimulated by the return electrode simultaneously with the rTPJ. The specific contribution of the rTPJ remains thus unclear in these two studies. In our study, we used a larger reference electrode that might be considered as “passive” so that the sole active electrode was placed over the rTPJ. This prevented us from measuring combined effects of the stimulation and return electrodes. If the rTPJ plays a causal role in the processing of mitigating circumstances to disculpate the agent, we should find a stronger effect of the rTPJ stimulation for the accidental harm compared to the attempted harm scenarios and compared to the neutral or intentional harm scenarios.

## Experiment 1

### Materials and Methods

#### Participants

Fifty-one students participated in this study in return of a small honorarium. Data from three participants were removed because of technical or human failures (leaving 16 participants in each group). All 48 remaining participants (27 female, mean age 22.33 ± 2.41 (*SD*), range 18–29 years) were healthy volunteers without any known psychiatric or neurological disorder and no contraindications to tDCS. They were all right handed according to the Edinburgh Handedness Inventory (Oldfield, 1971). They had normal or corrected to normal vision and spoke French fluently. Written informed consent was obtained. Participants were assigned following a random double blind procedure^1^ to one of the three experimental conditions (anodal, cathodal, and sham). The three groups were equivalent in terms of age (*F* < 1) and gender (χ^2^ < 1). During post-experiment briefing, participants did not report any expectations that were in line with our hypotheses. This study was carried out in accordance with the recommendations of the Commission d’Ethique Biomédicale Hospitalo-Facultaire de la Faculté de Médecine de l’UCL (registration number: B403201214597) with written informed consent from all participants.

#### Transcranial Direct Current Stimulation

The stimulation was delivered with a constant direct current stimulator (HDC-stim, Newronika, Milan, Italy) connected to two sponge electrodes. The 25 cm^2^ stimulation electrode was placed on the rTPJ over CP6 (same location as Santiesteban et al., 2012) according to the 10–20 EEG international system (Sharbrough et al., 1991; Herwig et al., 2003). The 51 cm^2^ reference electrode was placed over C3. A large reference electrode has been demonstrated to be functionally inert without diminishing the efficacy of the tDCS under the stimulation electrode (Nitsche et al., 2008). A current intensity of 0.8 mA was used for 20 min of stimulation (10 min oﬄine stimulation – 10 min online stimulation). With these parameters, the current density was 0.032 mA/cm^2^ and the total charge was 0.038 C/cm^2^. For the anodal and cathodal stimulations, the current ramped up until 100% intensity in 7 s. Once 100% of intensity was reached, the current remained constant until the end of the treatment. For the sham stimulation, the setup was the same as in the two other groups except that the current was turned off after it had ramped up to 100% intensity in the first 7 s. Thus, participants in the sham group also felt the initial itching sensation but received no active current for the rest of the stimulation. This method should be sufficient to keep participants blind to the stimulation condition (Gandiga et al., 2006; Ambrus et al., 2012).

#### Material

The verbal vignettes used in this study were inspired from those used by Young et al. (2007) and Cushman (2008). In adapting the vignettes, we followed recent recommendations to control for various parameters which can bias moral judgments (see Christensen and Gomila, 2012). The vignettes were presented in a standardized structure: one sentence presenting the context, one sentence presenting the protagonist’s belief and action (on the basis of which his intention to harm or not could be inferred) and one sentence with the action consequence (i.e., whether it caused or not harm to another person). Both protagonists (the agent and the victim) were always described in the third-person perspective (rather than placing participants in the role of one of the protagonists), they both had the same gender, they had names of equivalent frequency in the Belgian population and their relationship was not defined (control for kinship/friendship effect). The harm always occurred by action (never by omission), and there was never a self-benefice for the agent-protagonist. Furthermore, the harm occurred in the absence of physical contact between the two protagonists (control for the directness of harm) and the harm was always a physical injury (control for the kind of transgression). We also used familiar contexts to encourage everyday life moral judgments.

The experimental design consisted of an orthogonal manipulation of two factors, the intention to harm (no intention to harm vs. intention to harm) and the outcome of the action (neutral outcome vs. harmful outcome). Sixty-four contexts were created for the vignettes and each of these contexts was narrated in four different ways in order to conform to the four conditions of the 2 × 2 design (“neutral scenarios”: no intention to harm – neutral outcome; “accidental harm scenarios”: no intention to harm – harmful outcome; “attempted harm scenarios”: intention to harm – neutral outcome; “intentional harm scenarios”: intention to harm – harmful outcome; see **Table 2**), yielding a total of 256 vignettes. From these 256 vignettes, four lists were created so that each list included one of the 64 contexts and 16 vignettes per condition (16 neutral scenarios, 16 accidental harm scenarios, 16 attempted harm scenarios, 16 intentional harm scenarios). The different lists were equivalent in terms of number of words. Each list was further subdivided into two blocks: 32 vignettes to be presented before the tDCS stimulation (pre-stimulation condition) and 32 vignettes to be presented during and after the tDCS stimulation (post-simulation condition), with an equal number of vignettes (*n* = 8) per condition. The set of vignettes presented before and after stimulation was counterbalanced across participants. The full set of scenarios can be freely accessed via the following link: https://dx.doi.org/10.6084/m9.figshare.3427853.

**Table 2:** Examples of scenarios used in Experiments 1 and 2.

	Neutral Intention	Harmful Intention
**EXPERIMENT 1**		
Neutral Outcome	*Neutral scenario:* Steve and Nathan work in a shop. They restock the new merchandises on the shelves in the storehouse. Steve fills the top shelves while Nathan puts the products just below. Steve thinks that the shelf **will not break** under the weight of the boxes. Steve puts the box on it. The shelf **does not break** and Nathan is **OK.**	*Attempted Harm scenario:* Steve and Nathan work in a shop. They restock the new merchandises on the shelves in the storehouse. Steve fills the top shelves while Nathan puts the products just below. Steve thinks that the shelf **will break** under the weight of the boxes. Steve puts the box on it. The shelf **does not break** and Nathan is **OK.**
Harmful Outcome	*Accidental Harm scenario:* Steve and Nathan work in a shop. They restock the new merchandises on the shelves in the storehouse. Steve fills the top shelves while Nathan puts the products just below. Steve thinks that the shelf **will not break** under the weight of the boxes. Steve puts the box on it. The shelf **breaks** and Nathan is **hurt**.	*Intentional Harm scenario:* Steve and Nathan work in a shop. They restock the new merchandises on the shelves in the storehouse. Steve fills the top shelves while Nathan puts the products just below. Steve thinks that the shelf **will break** under the weight of the boxes. Steve puts the box on it. The shelf **breaks** and Nathan is **hurt.**
**EXPERIMENT 2**		
Neutral Outcome	*Neutral scenario:* Steve and Nathan work in a shop. They restock the new merchandises on the shelves in the storehouse. Steve fills the top shelves while Nathan puts the products just below. Steve puts the box on it. Steve thought that the shelf **would not break** under the weight of the boxes. The shelf **does not break** and Nathan is **OK.**	*Attempted Harm scenario:* Steve and Nathan work in a shop. They restock the new merchandises on the shelves in the storehouse. Steve fills the top shelves while Nathan puts the products just below. Steve puts the box on it. Steve thought that the shelf **would break** under the weight of the boxes. The shelf **does not break** and Nathan is **OK.**
Harmful Outcome	*Accidental Harm scenario:* Steve and Nathan work in a shop. They restock the new merchandises on the shelves in the storehouse. Steve fills the top shelves while Nathan puts the products just below. Steve puts the box on it. Steve thought that the shelf **would not break** under the weight of the boxes. The shelf **breaks** and Nathan is **hurt**.	*Intentional Harm scenario:* Steve and Nathan work in a shop. They restock the new merchandises on the shelves in the storehouse. Steve fills the top shelves while Nathan puts the products just below. Steve puts the box on it. Steve thought that the shelf **would break** under the weight of the boxes. The shelf **breaks** and Nathan is **hurt.**

#### Experimental Design and Procedure

The vignettes were presented in a pseudorandom order using PsychoPy 1.76.00 (Peirce, 2007, 2009), with the conditions counterbalanced across blocks and subjects. We used a similar procedure as the one proposed by Greene et al. (2001) in order to control for the moment in which the participant was exposed to each piece of information in the scenario. The vignettes were presented in three cumulative segments (previous segments remained on the screen when later segments were added): (1) the contextual information (12 s), (2) the protagonist’s belief and action (an additional 8 s), (3) the outcome (an additional 4 s). All of the story text was then removed from the screen and replaced with the question and the horizontal response scale. Subjects had 7 s to judge “How much should the agent’s behavior be blamed?” (“A quel point est-ce blamable de se comporter comme l’agent?”) on the 7-points response scale ranging from 1 “Not at all” (“Pas du tout”) to 7 “Very much” (“Tout à fait”).

**FIGURE 1:**
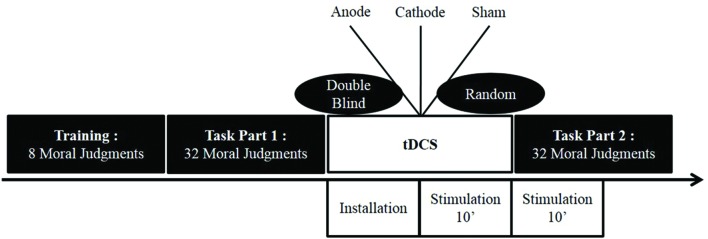
**Illustration of the tDCS protocol used in Experiments 1 and 2**.

The first block of vignettes was presented without any stimulation (baseline). There was then a 20-min break, with the first 10 min of the break used for the electrode montage and the next 10 min to start the tDCS stimulation. The second block of vignettes was then presented with only the first 10 min still under tDCS stimulation (anodal, cathodal, and sham; see **Figure 1** for an illustration of the tDCS stimulation timing).

### Results

#### Factors Affecting Blame Ratings at Baseline

In a first analysis, we examined the factors influencing participant’s blame ratings prior to any tDCS. We conducted a repeated measure ANOVA on the ratings at baseline without distinguishing the groups and with Intention (intention to harm vs. no intention to harm) and Outcome (harmful outcome vs. neutral outcome) as within-subject factors.

We found a significant main effect of Intention [*F*_(1,47)_ = 316.689, *p* < 0.001, $\eta_{p}^{2}$ = 0.871], with intention to harm scenarios (mean 5.31 ± SE 0.12) being judged more blameworthy than no intention to harm scenarios (2.75 ± 0.10) and, a significant main effect of Outcome [*F*_(1,47)_ = 70.484, *p* < 0.001, $\eta_{p}^{2}$ = 0.600], with harmful outcome scenarios (4.58 ± 0.08) being judged more blamable than neutral outcome scenarios (3.48 ± 0.13). The Intention × Outcome interaction was not significant [*F*_(1,47)_ = 0.378, *p* = 0.542, $\eta_{p}^{2}$ = 0.008].

Note that a further analysis with tDCS condition (anode vs. cathode vs. sham) as additional between-subject factor showed that the effects mentioned above where present in all three groups prior to the start of stimulation. Indeed, there was no significant tDCS condition interaction [all *F*_(2,45)_ < 2.346, all *p* > 0.107, all $\eta_{p}^{2}$ < 0.094].

#### Modulation of Blame Judgments as a Function of tDCS Condition

In order to examine changes in ratings as a consequence of tDCS, we conducted a repeated measure ANOVA on the ratings with Intention (intention to harm vs. no intention to harm), Outcome (harmful outcome vs. neutral outcome), and Time (pre-stimulation vs. post-stimulation) as within-subject factors and with tDCS Condition (anode vs. cathode vs. sham) as between-subject factor. Any interaction effects involving both Time and tDCS Condition were of particular interest. No such interaction effect was statistically significant [all *F*_(2,45)_ < 2.007, all *p* > 0.146, all $\eta_{p}^{2}$ < 0.082].

**FIGURE 2:**
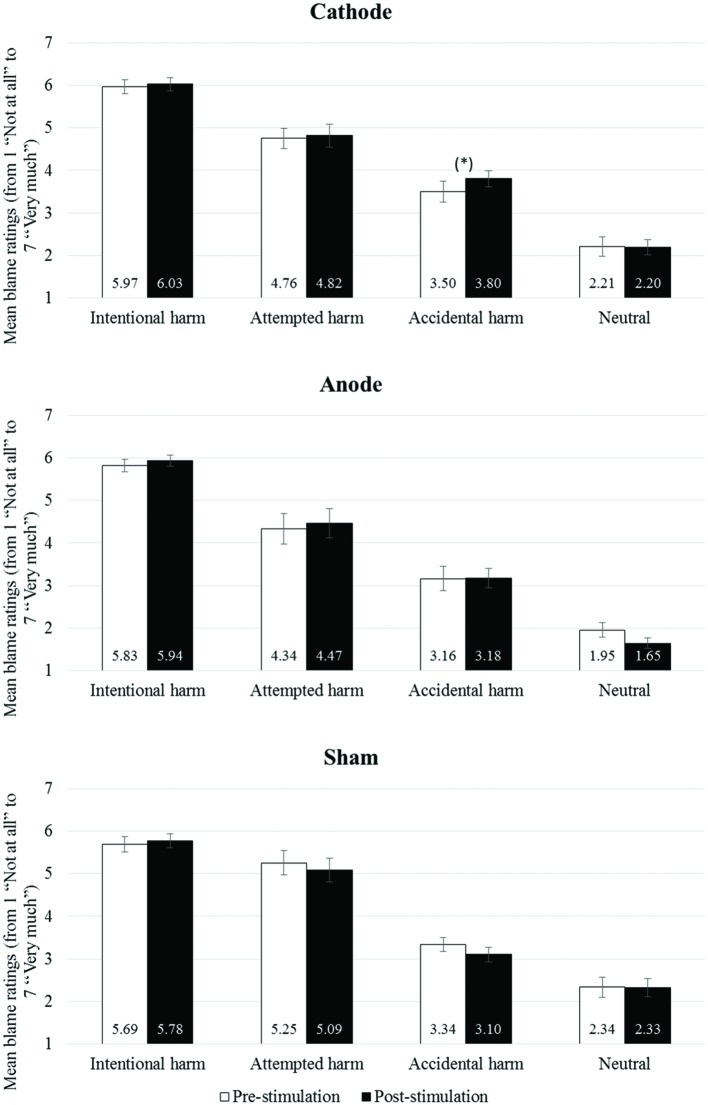
**Mean blame ratings (from 1 “Not at all” to 7 “Very much”) as a function of moral scenarios (Intentional harm scenarios, Attempted harm scenarios, Accidental harm scenarios and Neutral scenarios) and time (Pre-stimulation and Post-stimulation) for the Cathodal Group, the Anodal Group, and the Sham Group.** Error bars represent standard mean error. (*)*p* < 0.10; *p < 0.05; **p < 0.01.

Nevertheless, to explore the data, we conducted pairwise comparisons comparing pre-stimulation with post-stimulation ratings for each of the four types of scenarios (neutral scenarios, accidental harm scenarios, attempted harm scenarios, and intentional harm scenarios). The analyses showed a marginally significant effect (at an uncorrected level for multiple comparisons) for one experimental condition, namely the accidental harm condition [*t*_(15)_ = -2.080, *p* = 0.055, *d* = 0.564], with a marginally significant increase in the severity of blame rating following tDCS cathodal stimulation (pre-stimulation: 3.50 ± 0.24; post-stimulation: 3.80 ± 0.18). No effect of Time (pre- compared to post-stimulation) reached the significance level in the anodal stimulation condition [all *t*_(15)_ < 1.848, all *p*> 0.084, all *d* < 0.472] or the sham stimulation condition [all *t*_(15)_ < 1.266, all *p* > 0.225, all *d* < 0.328; **Figure 2**].

### Conclusion and Discussion

The results of Experiment 1 showed a marginal effect (1) of cathodal stimulation only and (2) on accidental harm scenarios only. This pattern of result is in line with the idea that the rTPJ may play a role in the consideration of mitigating circumstances when attributing moral responsibility. The effect was, however, only marginally significant and could either reflect a lack of power (due to sample size or current density) or could be a false positive. We thus conducted a follow-up experiment with an improved design.

## Experiment 2

Experiment 2 was designed to replicate the results of Experiment 1 while improving the design. Firstly, we shortened the response time (from 7 to 4 s) to better capture initial judgments. Secondly, we clarified some sentences to avoid ambiguities and changed the position of the action information in the scenario. In Experiment 1, the action information was presented at the end of the intention segment while in Experiment 2, the action information was presented at the end of the context segment, this minor change was done to facilitate the inference of the agent’s intention. Thirdly, we used a punishment question (“How much punishment tokens would you give to the agent?”) as it appeared that participants did not all interpret the “blame” question in the same way (some construed it as a punishment judgment others as a wrongness judgment). Moreover, using a punishment question increases the need to take into account the outcome. Indeed, punishment assignment is thought to occur after the attribution of moral responsibility (or blame) and requires integrating the amount of harm caused (Buckholtz et al., 2015). When an attempt to harm failed compared to when it succeeds, the amount of punishment deserved should be mitigated by the fact that no harm really occurred. An absence of tDCS on attempted harms even though a punishment question is used would thus also provide indirect support for the hypothesis that it is not the rTPJ but other brain areas which sustain such different form of mitigation. Thirdly, we now explicitly asked participants to respond with their right hand on a vertical response scale in order to avoid any spatial effect due to the temporo-parietal brain area stimulation. Finally, we increased the intensity of the tDCS from 0.8 to 1.5 mA.

### Materials and Methods

#### Participants

Seventy-five new right-handed students participated in this study for a small honorarium. Data from three participants were removed because of technical or human failures (leaving 24 participants in each group). The recruitment procedure was the same as for the study 1. All 72 remaining participants [half female, mean age 21.65 ± 1.75 (*SD*), range 18–26 years] were assigned following a random double blind procedure to the tDCS conditions (anodal, cathodal, and sham). Groups did not differ significantly in terms of age [*F*_(2,69)_ = 2.901, *p* = 0.062] and gender. Again, during the post-experiment briefing, participants did not report any expectations that were in line with our hypotheses. We followed the same ethical protocol as in Experiment 1.

#### Transcranial Direct Current Stimulation

The procedure was exactly the same as for Experiment 1 with the only exception that the current intensity was raised to 1.5 mA. With this new parameter, the current density was 0.06 mA/cm^2^ and the total charge was 0.072 C/cm^2^.

#### Material

We used the same scenarios as the ones used in Experiment 1 (except for the changes mentioned above, see **Table 2** for examples). In addition, we conducted a pretest to collect ratings about the severity of the harm resulting from the actions used in our design. Thirty-three participants were asked to respond to the question “The consequences for the victim are …?” (“Les conséquences pour la victime sont …?”) on a scale from 0 “Not serious at all” (“Pas graves du tout”) to 6 “Very serious” (“Très graves”). We then averaged the scores for each scenario and we split the 64 scenarios according to the median value (3.48); 33 scenarios were below the median split and were considered as low harm severity scenarios and 31 scenarios were above the median split and were considered as high harm severity scenarios. Within each list of scenarios, harm severity was evenly distributed across the different experimental conditions. The full set of scenarios can be freely accessed via the following link: https://dx.doi.org/10.6084/m9.figshare.3427853.

#### Experimental Design and Procedure

The design and the procedure were the same as in the Experiment 1 except for the changes mentioned above and the use of an upgraded version of PsychoPy (1.78.00; Peirce, 2007, 2009) for the stimuli presentation. Each participant was presented with one version of each moral scenario and was asked to judge the number of punishment tokens they would give to the agent. After having read each scenario, subjects had 4 s to judge “How much punishment tokens would you give to the agent?” (“Combien de jetons de punition donneriez-vous à l’agent?”) on 7-points response vertical scale from 0 punishment token to 6 punishment tokens.

### Results

#### Factors Affecting Punishment Ratings at Baseline

In a first analysis, we examined the factors influencing participant’s punishment ratings prior to any tDCS. We conducted a repeated measure ANOVA on the ratings at baseline without distinguishing the groups and with Intention (intention to harm vs. no intention to harm), Outcome (harmful outcome vs. neutral outcome), and Harm Severity (high harm severity vs. low harm severity) as within-subject factors.

We found a significant main effect for all three factors: Intention [*F*_(1,71)_ = 354.535, *p* < 0.001, $\eta_{p}^{2}$ = 0.833], Outcome [*F*_(1,71)_ = 231.171, *p* < 0.001, $\eta_{p}^{2}$ = 0.765] and Harm Severity [*F*_(1,71)_ = 57.811, *p* < 0.001, $\eta_{p}^{2}$ = 0.449], with more severe punishment attributed when the intention was to harm (3.68 ± 0.14) rather than neutral (0.97 ± 0.08), when harm occurred (3.01 ± 0.09) compared to no harm occurred (1.65 ± 0.10) and when the harm (really or potentially) caused was high (2.53 ± 0.08) rather than low (2.12 ± 0.09).

We also found two significant interactions. First, the Intention by Outcome interaction was significant [*F*_(1,71)_ = 9.084, *p* = 0.004, $\eta_{p}^{2}$ = 0.113]. Pairwise comparisons with Bonferroni correction showed a significant effect of Intention both for harmful outcome scenarios (*p* < 0.001; intention to harm: 4.47 ± 0.13, no intention to harm: 1.55 ± 0.11) and for neutral outcome scenarios (*p* < 0.001; intention to harm: 2.90 ± 0.17, no intention to harm: 0.39 ± 0.60). This interaction can be explained by a stronger effect of intention when the outcome was harmful than when the outcome was neutral. The difference between the punishment ratings of the intentional harm (mean = 4.47) and the accidental harm (mean = 1.55) conditions was 2.92 and numerically higher than the difference between the punishment ratings of the attempted harm (mean = 2.90) compared to the neutral conditions (mean = 0.39) which was 2.51. Pairwise comparison with Bonferroni correction also showed a significant effect of Outcome both for the intention to harm condition (*p* < 0.001; harmful outcome: 4.47 ± 0.13, neutral outcome: 2.90 ± 0.17) and for the no intention to harm condition (*p* < 0.001; harmful outcome: 1.55 ± 0.11, neutral outcome: 0.39 ± 0.60). Here, the interaction can be explained by a stronger effect of the outcome when there was an intention to harm than when there was no intention to harm. The difference between the punishment ratings in the intentional harm (mean = 4.47) and the attempted harm conditions (mean = 2.90) was 1.57 and numerically higher than the difference between the punishment ratings in the accidental harm (mean = 1.55) compared to the neutral conditions (mean = 0.39) which was 1.16.

The second significant interaction was between Harm Severity and Outcome [*F*_(1,71)_ = 10.472, *p* = 0.002, $\eta_{p}^{2}$ = 0.129]. Pairwise comparison with Bonferroni correction showed a significant effect of Harm Severity both for the harmful outcome condition (*p* < 0.001; high harm severity: 3.29 ± 0.10, low harm severity: 2.72 ± 0.11) and for the neutral outcome condition (*p* < 0.001; high harm severity: 1.78 ± 0.10, low harm severity: 1.52 ± 0.10). The interaction can be explained by a stronger effect of harm severity for the harmful outcome than for the neutral outcome condition. Indeed, the difference between the punishment ratings in the harmful outcome with high harm severity (mean = 3.29) and the harmful outcome with low harm severity (mean = 2.72) was 0.57, which is numerically higher than the difference between the punishment ratings in the neutral outcome with high harm severity (mean = 1.78) and the neutral outcome with low harm severity (mean = 1.52) which was 0.26. Pairwise comparison with Bonferroni correction also showed a significant effect of Outcome both for high harm severity scenarios (*p* < 0.001; harmful outcome: 3.29 ± 0.10, neutral outcome: 1.78 ± 0.10) and for low harm severity scenarios (*p* < 0.001; harmful outcome: 2.72 ± 0.11, neutral outcome: 1.52 ± 0.10). The interaction can be explained by a stronger effect of the outcome for the high harm severity than for the low harm severity. The difference between the punishment ratings in the harmful outcome with high harm severity (mean = 3.29) and the neutral outcome with high harm severity (mean = 1.78) was 1.51 and numerically higher than the difference between the punishment ratings in the harmful outcome with low harm severity (mean = 2.72) and the neutral outcome with low harm severity (mean = 1.52) which was 1.20.

Finally, the interaction between Intention and Harm Severity was not significant [*F*_(1,71)_ = 3.459, *p* = 0.067, $\eta_{p}^{2}$ = 0.046]. The triple interaction was not significant either [*F*_(1,71)_ = 3.068, *p* = 0.084, $\eta_{p}^{2}$ = 0.041].

Note that additional analysis with tDCS Condition (anode vs. cathode vs. sham) as between-subject factor showed that the effects mentioned above where present in all three groups prior to the start of stimulation. Indeed, there was no significant tDCS Condition interaction [all *F*_(2,69)_ < 2.114, all *p* > 0.128, all $\eta_{p}^{2}$ < 0.058].

#### Modulation of Punishment Judgments as a Function of tDCS Condition

In order to examine changes in ratings as a consequence of tDCS, we conducted a repeated measure ANOVA on the ratings with Intention (intention to harm vs. no intention to harm), Outcome (harmful outcome vs. neutral outcome), Harm Severity (low harm severity vs. high harm severity), and Time (pre-stimulation vs. post-stimulation) as within-subject factors and with tDCS Condition (anode vs. cathode vs. sham) as between-subject factor. Again, any interaction effect involving both Time and tDCS Condition were of particular interest.

The only such interaction effect which was sufficiently close to the statistical significance level to be considered, was the four-way Intention by Outcome by Time by tDCS Condition interaction [*F*_(2,69)_ = 3.123, *p* = 0.050, $\eta_{p}^{2}$ = 0.083]. To explore this interaction further, we conducted separate repeated measure ANOVAs for each tDCS Condition with Intention, Outcome and Time as within-subject factors. Any main effect or interaction involving Time was of particular interest.

**FIGURE 3:**
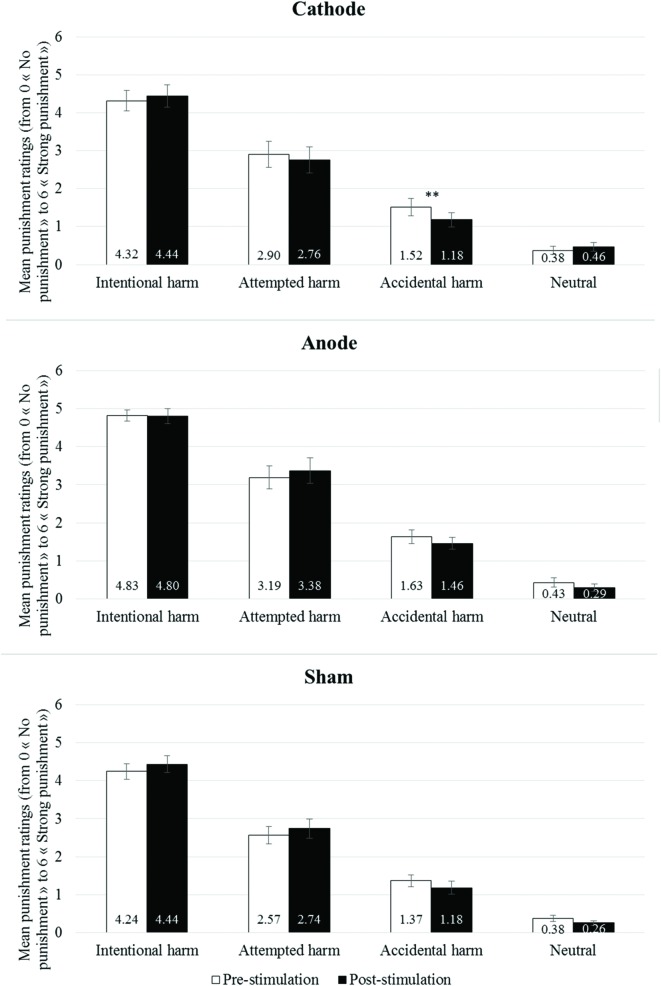
**Mean number of punishment tokens (from 0 “No punishment” to 6 “Strong punishment”) as a function of moral scenarios (Intentional harm scenarios, Attempted harm scenarios, Accidental harm scenarios, and Neutral scenarios) and time (Pre-stimulation and Post-stimulation) for the Cathodal Group, the Anodal Group, and the Sham Group.** Error bars represent standard mean error. (*)*p* < 0.10; *p < 0.05; **p < 0.01.

In the anodal stimulation condition, no effect involving Time reached the significance level [all *F*_(1,23)_ < 2.107, all *p* > 0.160, all $\eta_{p}^{2}$ < 0.084]. In the cathodal stimulation condition, there was a significant three way Intention by Outcome by Time interaction effect [*F*_(1,23)_ = 9.463; *p* = 0.005, $\eta_{p}^{2}$ = 0.292]. Paired-wise comparisons comparing pre-stimulation with post-stimulation ratings for each of the four types of scenarios (neutral scenarios, accidental harm scenarios, attempted harm scenarios, and intentional harm scenarios) showed that the ratings only changed for one type of scenarios, namely the accidental harm scenarios [*t*_(23)_ = 3.076, *p* = 0.005, *d* = 0.677], with a significance decrease in the severity of punishment rating following tDCS stimulation (pre-stimulation: 1.52 ± 0.23; post-stimulation: 1.18 ± 0.19). This effect of cathodal stimulation on the accidental harm scenarios remained significant after applying a Bonferroni correction for multiple comparisons (the adjusted threshold for significance in case of six paired-wise comparisons is 0.05/6 = 0.008). In the sham condition, there was a significant Intention by Time interaction effect [*F*_(1,23)_ = 5.685, *p* = 0.026, $\eta_{p}^{2}$ = 0.198], however, none of the paired-wise comparisons comparing pre-stimulation with post-stimulation ratings reached significance [intention to harm : *t*_(23)_ = -1.644, *p* = 0.114, *d* = 0.335; no intention to harm : *t*_(23)_ = 1.757, *p* = 0.092, *d* = 0.350; **Figure 3**].

### Conclusion and Discussion

Again, an effect of tDCS was observed in only one stimulation condition (i.e., cathodal) and only one type of scenarios (i.e., accidental harms scenarios), with a highly significant decrease in the severity of judgments of accidental harms under higher cathodal current density. This effect of Time observed in the cathodal stimulation was specific to the stimulation and not just a general effect of Time.

## General Discussion

Across two experiments, we found that cathodal tDCS applied to the rTPJ specifically modulated the moral judgments of accidental harms; judgments of attempted harms were not affected. A marginal increase in the severity of judgments of accidental harms was found under low cathodal current density (Experiment 1; but the effect did not reach the statistical significance level) and a highly significant decrease in the severity of such judgments was found under higher cathodal current density (Experiment 2). On the other hand, anodal tDCS did not significantly modulate moral judgments (**Table 3**). We discuss these results in relation to the role of the rTPJ in moral cognition and in relation to methodological aspects regarding the use of tDCS in moral cognition.

**Table 3:** Summary of the stimulation effects on moral judgments found in Experiments 1 and 2.

	Type of TPJ stimulation	Question asked	Stimulation parameters	Intentional harm scenarios (intention to harm and a harmful outcome)	Attempted harm scenarios (intention to harm but no harmful outcome)	Accidental harm scenarios (no intention to harm but a harmful outcome)	Neutral scenarios (no intention to harm and no harmful outcome)
Leloup et al. (present paper)	Experiment 1: Decreased excitability of rTPJ (cathodal tDCS)	“How much should the agent’s behavior be blamed?” on a scale from 1 (Not at all) to 7 (Very much).	**Stimulation electrode** **Size:** 25 cm^2^ **Location:** CP6 **Reference electrode** **Size:** 51 cm^2^ **Location:** C3 **Intensity:** 0.8 mA **Duration:** 20’ (10’ oﬄine – 10’ online) **Current density:** 0.032 mA/cm^2^ **Total charge:** 0.038 C/cm^2^*	No effect	No effect	A trend to be judged as more blamable	No effect
	Experiment 2: Increased excitability of rTPJ (cathodal tDCS)	“How much punishment tokens would you give to the agent?” on a scale from 0 punishment token to 6 punishment tokens.	**Stimulation electrode** **Size:** 25 cm^2^ **Location:** CP6 **Reference electrode** **Size:** 51 cm^2^ **Location:** C3 **Intensity:** 1.5 mA **Duration:** 20’ (10’ oﬄine – 10’ online) **Current density:** 0.06 mA/cm^2^ **Total charge:** 0.072 C/cm^2^*	No effect	No effect	Judged as less punishable	No effect

*Total charge has been calculated according to the formula used by Nitsche et al. (2003a): [intensity (A)/electrode size (cm^2^)] × total stimulation duration (s).

### What Is the Role of the rTPJ in Moral Cognition?

While previous studies that investigated the role of the rTPJ in moral cognition focused on the role of this brain region in belief processing, in our study we examined the potential additional role in processing the mitigating circumstances that reduce the moral responsibility of someone who committed a harmful action. Indeed, it has been recently proposed that the rTPJ may be key in this particular important aspect of moral reasoning (Buckholtz et al., 2008, 2015; Young and Saxe, 2009; Buckholtz and Marois, 2012; Koster-Hale et al., 2013). However, so far the empirical evidence has only come from fMRI studies and the link has been only correlational. Across the four types of moral scenarios that we used in our study, there was only one type that included circumstances which can reduce someone’s moral responsibility (i.e., our accidental harm scenarios in which an agent unintentionally harmed another person). Quite strikingly, in both our experiments, this was the only type of scenarios in which participants’ judgments were affected by the rTPJ stimulation. Our results are thus in line with the idea that the rTPJ plays a causal role in the processing of mitigating circumstances when attributing moral responsibility.

Are there alternative explanations for our results? The first alternative to be considered is that the differential pattern of stimulation effect that we observed across the accidental and attempted harm scenarios results from a combination of ceiling and floor effects as proposed by Sellaro et al. (2015). As a reminder, Sellaro et al. (2015) proposed that the rTPJ is key in processing the false beliefs of the protagonist but that the stimulation of the rTPJ can only manifest itself as a facilitation of belief processing in the accidental harm scenarios under anodal stimulation and as a reduction of belief processing in the attempted harm condition under cathodal stimulation or “inhibitory” TMS. This ceiling/floor effect hypothesis does not account very well for our results as (1) we find both an increase (Experiment 1) and a decrease (Experiment 2) of the reliance on the protagonist’s harmless intention for the same accidental harm scenarios and (2) no significant effect of stimulation for the attempted harm scenarios with either cathodal or anodal stimulation.

Both, the accidental and the attempted harm scenarios that we used required to reason about beliefs and desires to infer the protagonist’s intention. A second alternative explanation is that the processing of beliefs and/or desires was harder in the accidental than the attempted harm scenarios and that we thus only observed a stimulation effect in the most demanding condition. In terms of belief processing and given the way our scenarios were constructed, it seems highly unlikely that realizing that the protagonist’s belief was false was harder in the accidental than the attempted harm scenarios. In terms of desire processing, one could even argue that the mentalizing demands may have been higher in the attempted than the accidental harm scenarios as in the attempted harm scenarios there was a stronger discrepancy between the protagonist’s desire (to harm) and participants’ desire if they had been in the same situation (we can indeed assume that most participants adhered to the moral norm of not hurting other people as demonstrated by their higher blame and punishment ratings when the protagonist had an intention to harm). Thus, there seems no obvious reason why belief or desire reasoning would have been harder in the accidental than the attempted harm scenarios.

Overall, it seems rather that it is the processing of intention and its integration in moral judgment that best explains the difference between accidental and attempted harm scenarios. In the case of attempted harm scenarios, there is no information available that could reduce the protagonist’s moral responsibility since participants can only come to the conclusion that the protagonist intended to harm someone. Thus even if during the reading of the attempted harm scenarios, participants were looking for mitigating circumstances, none were to be found. Increasing or decreasing the efficiency of the search for and integration of mitigating circumstances could thus not impact on participants’ blame or punishment ratings of attempted harms. In contrast, in the accidental harm scenarios, the protagonist’s false belief constitutes a mitigating circumstance. Here increasing or decreasing the efficiency of the search for and integration of mitigating circumstances should indeed directly impact on the blame or punishment ratings. It is interesting to note that the rTPJ does not seem to play a general role in adjusting moral judgment according to available circumstances. Judgments of intended harms that failed (i.e., attempted harms) are usually adjusted compared to intended harms that succeeded (i.e., intentional harms) in terms of punishment because no actual harm occurred in the former. Buckholtz et al. (2015) recently showed that the adjustment in moral judgment linked to the amount of harm caused to the victim can be causally linked to the DLPFC. In line with this, no effect of rTPJ stimulation was found on attempted harms in our study.

While our results are compatible with a role of the rTPJ in processing the mitigating circumstances that can reduce someone’s moral responsibility (such as mental illness or accidental harm), we do not want to claim that this is the sole role of the rTPJ in moral cognition. The contribution of the rTPJ in moral judgment is probably multiple and includes the processing of the beliefs and desires of the persons involved. The potential to measure each contributing role of the rTPJ may, however, depend on the specific design and methods used in a study. For example, in our study the harmful outcomes were less severe than in previous studies (unlike the scenarios used in previous studies, Young et al., 2010a; Sellaro et al., 2015; Ye et al., 2015, scenarios used in our experiments never resulted in someone’s death). In case of severe harmful outcomes, participants may be more reluctant to consider mitigating circumstances, thereby preventing the observation of a change in the efficiency of mitigating circumstances processing following rTPJ tDCS. Secondly, our choice of punishment and blame questions rather than a permissibility question (all previous studies used a permissibility question) may have put more weight on the search for mitigating circumstances. Indeed, searching for mitigating circumstances is particular important for establishing the moral responsibility of an agent, and moral responsibility is particularly important to decide the extent to which the agent should be blamed or punished (Buckholtz et al., 2015). It is possible that other types of questions and/or scenario characteristics would recruit more the processing of the agents’ beliefs and intentions than the processing of mitigating circumstances. In the latter case, depending on the possible existence of ceiling/floor effects discussed by Sellaro et al. (2015), rTPJ tDCS would either affect judgments of attempted or accidental harm scenarios rather than both types of scenarios. It would be important to investigate the impact of the questions and scenarios characteristics in future research.

### Methodological Aspect about the Use of tDCS in Moral Cognition

In our two experiments, we only found significant effects under cathodal stimulation. This is surprising given that in most previous studies which used tDCS to investigate mentalizing, cathodal stimulation showed no effect (e.g., Santiesteban et al., 2012; Sellaro et al., 2015). Some authors noticed that the effects of cathodal stimulation are less consistent for higher-order cognitive functions (Jacobson et al., 2012). When considering more largely the literature on the effects of tDCS on social cognition, it is in fact not unusual to find the modulation of social behavior following either cathodal (e.g., Karim et al., 2010; Mai et al., 2016) or anodal stimulation (e.g., Priori et al., 2008; Santiesteban et al., 2012; Kuehne et al., 2015; Sellaro et al., 2015) but not both. Some studies showed that gender may influence which type of stimulation is effective (Fumagalli et al., 2010) and others have highlighted the possible role of ceiling effects in cortical excitability (Karim et al., 2010; Sellaro et al., 2015). This clearly needs further investigation.

In our two experiments, the cathodal stimulation had opposite behavioral effects possibly depending on the current intensity and hence the current density. In Experiment 1, lower intensity cathodal stimulation led participants to make harsher judgments consistent with the idea that they took less into account the mitigating circumstances and that the stimulation may have reduced the cortical excitability of the rTPJ. On the other hand, in Experiment 2, higher intensity cathodal stimulation led participants to make more lenient judgments consistent with the idea that they took the mitigating circumstances more into account and that the stimulation may have increased the cortical excitability of the rTPJ. A generally accepted idea is that cathodal stimulation decreases cortical excitability (e.g., Nitsche et al., 2003b, 2008; Jacobson et al., 2012). However, new evidence suggests that increasing the intensity or the duration of cathodal stimulation, amongst other things, can induce shifts in cortical excitability and lead cathodal stimulation to have facilitatory effects (Batsikadze et al., 2013; Pirulli et al., 2014). Our data are in line with these results as we changed the intensity and hence the density of the direct current across our two experiments: in Experiment 1, the current intensity was 0.8 mA (current density: 0.032 mA/cm^2^; total charge: 0.038 C/cm^2^) while in Experiment 2, the current intensity was raised to 1.5 mA (current density: 0.06 mA/cm^2^; total charge: 0.072 C/cm^2^). For future research, it would be important to keep in mind that enhancing the intensity of cathodal stimulation can shift the cortical excitability. Putting together the findings from Santiesteban et al. (2012) and Sellaro et al. (2015) and the fact that in our study we only had effects after cathodal stimulation and that this effect was the strongest when it had enhanced the cortical excitability, the pattern of results fits with the idea that it is easier to enhance the cortical excitability of the rTPJ with tDCS than to reduce it.

In sum, the role of the rTPJ in moral cognition is probably multiple and here we show evidence for a causal role of the rTPJ in processing mitigating circumstances that can reduce someone moral responsibility when causing harm. In our study, the mitigating circumstance was the fact of causing harm unintentionally but we could expect similar effects in the case of other mitigating circumstances such as diminished mental capacity (Buckholtz et al., 2008, 2015). It is likely that the role of the rTPJ in moral cognition is not limited to this specific role and that depending on the experimental design one or the other types of role may be better brought to light. Future investigations of these multiple roles and how they depend on specific design parameters would help understand the seemingly discrepant patterns of results observed so far following rTPJ stimulation. tDCS seems a promising technique for such investigation.

## Author Contributions

LL participated in the design, data collection and data analysis of both experiments, and wrote the manuscript. DM participated in the design, data collection and data analysis of the first experiment, and commented on the written manuscript. GA participated in the data collection of the second experiment and commented on the written manuscript. YV participated in the design of both experiments and in the writing of the manuscript. DS participated in the design and data analysis of both experiments and in the writing of the manuscript.

## Conflict of Interest Statement

The authors declare that the research was conducted in the absence of any commercial or financial relationships that could be construed as a potential conflict of interest.

## Footnotes

1 We used the Newronika tDCS system which is made up of two devices, the programming device and the stimulator device. Two experimenters were involved in the experiments. One of the experimenters entered the stimulation parameters in the programming device and the other experiment used the stimulator device with participants. Thus, the experimenter in contact with the participants did not know the condition in which each participant was assigned to.
